# Pretherapeutic FDG-PET total metabolic tumor volume predicts response to induction therapy in pediatric Hodgkin’s lymphoma

**DOI:** 10.1186/s12885-018-4432-4

**Published:** 2018-05-03

**Authors:** Julian M. M. Rogasch, Patrick Hundsdoerfer, Frank Hofheinz, Florian Wedel, Imke Schatka, Holger Amthauer, Christian Furth

**Affiliations:** 1 Charité – Universitätsmedizin Berlin, Corporate Member of Freie Universität Berlin, Humboldt-Universität zu Berlin, and Berlin Institute of Health, Department of Nuclear Medicine, Augustenburger Platz 1, D-13353 Berlin, Germany; 2 Berlin Institute of Health, Department of Pediatric Oncology/Hematology, Berlin, Germany; 3 Berlin Institute of Health (BIH), Anna-Louisa-Karsch-Str. 2, Berlin, Germany; 4 PET Center, Helmholtz Zentrum Dresden-Rossendorf, Institute of Radiopharmaceutical Cancer Research, Dresden, Germany

**Keywords:** Pediatric Hodgkin’s lymphoma, Early response assessment, FDG-PET, Metabolic tumor volume, Asphericity

## Abstract

**Background:**

Standardized treatment in pediatric patients with Hodgkin’s lymphoma (HL) follows risk stratification by tumor stage, erythrocyte sedimentation rate and tumor bulk. We aimed to identify quantitative parameters from pretherapeutic FDG-PET to assist prediction of response to induction chemotherapy.

**Methods:**

Retrospective analysis in 50 children with HL (f:18; m:32; median age, 14.8 [4–18] a) consecutively treated according to EuroNet-PHL-C1 (*n* = 42) or -C2 treatment protocol (*n* = 8). Total metabolic tumor volume (MTV) in pretherapeutic FDG-PET was defined using a semi-automated, background-adapted threshold. Metabolic (SUVmax, SUVmean, SUVpeak, total lesion glycolysis [MTV*SUVmean]) and heterogeneity parameters (asphericity [ASP], entropy, contrast, local homogeneity, energy, and cumulative SUV-volume histograms) were derived. Early response assessment (ERA) was performed after 2 cycles of induction chemotherapy according to treatment protocol and verified by reference rating. Prediction of inadequate response (IR) in ERA was based on ROC analysis separated by stage I/II (1 and 26 patients) and stage III/IV disease (7 and 16 patients) or treatment group/level (TG/TL) 1 to 3.

**Results:**

IR was seen in 28/50 patients (TG/TL 1, 6/12 patients; TG/TL 2, 10/17; TG/TL 3, 12/21). Among all PET parameters, MTV best predicted IR; ASP was the best heterogeneity parameter. AUC of MTV was 0.84 (95%-confidence interval, 0.69–0.99) in stage I/II and 0.86 (0.7–1.0) in stage III/IV. In patients of TG/TL 1, AUC of MTV was 0.92 (0.74–1.0); in TG/TL 2 0.71 (0.44–0.99), and in TG/TL 3 0.85 (0.69–1.0). Patients with high vs. low MTV had IR in 86 vs. 0% in TG/TL 1, 80 vs. 29% in TG/TL 2, and 90 vs. 27% in TG/TL 3 (cut-off, > 80 ml, > 160 ml, > 410 ml).

**Conclusions:**

In this explorative study, high total MTV best predicted inadequate response to induction therapy in pediatric HL of all pretherapeutic FDG-PET parameters – in both low and high stages as well as the 3 different TG/TL.

**Trial registration:**

Ethics committee number: EA2/151/16 (retrospectively registered).

**Electronic supplementary material:**

The online version of this article (10.1186/s12885-018-4432-4) contains supplementary material, which is available to authorized users.

## Background

Hodgkin’s lymphoma (HL) accounts for about 7% of pediatric malignancies and about 1% of childhood cancer-related deaths in the United States [1]. Treatment intensity within standardized treatment protocols is based on different treatment groups/treatment levels (TG/TL) defined by the clinical stages I to IV, the presence of extranodal lesions, elevated erythrocyte sedimentation rate (ESR, ≥ 30 mm/h) or a tumor bulk ≥ 200 ml [2, 3]. F18-fluorodesoxyglucose-positron emission tomography/computed tomography (FDG-PET/CT) allows for combined functional-metabolic and morphological imaging both at initial staging to define the patient’s clinical stage [4] and during treatment to assess the presence or absence of remaining vital lymphoma tissue [5–7]. In particular, FDG-PET/CT has become essential for early response assessment (ERA) performed after 2 cycles of induction therapy [2, 3, 5]; only if FDG-avid tissue is still present in initially involved sites (semi-quantified as Deauville score > 3 or quantified with qPET [6]) or bulky lesions show < 50% volume reduction in CT, patients in all TG/TL are currently eligible for radiotherapy (RT) dependent on late response assessment following further chemotherapy. In patients with adequate response (AR), RT can be omitted irrespective of the TG/TL – and late effects due to radiotherapy, especially secondary malignancies, are avoided [8, 9]. Nevertheless, prediction of OS by ERA may inferior to late response assessment (LRA) [10]. Furthermore, initial prediction of response to induction therapy could even allow individualized treatment intensification to prevent IR a priori.

Despite this important role of FDG-PET/CT, no initial PET based parameter has been identified so far to predict response at ERA in pediatric HL patients – including the standardized uptake value (SUV) as the most common quantitative FDG-PET parameter [11]. Nevertheless, Meignan et al. recently showed in adult patients with follicular lymphoma (mostly stage III or IV disease) that a high initial metabolic tumor volume (MTV) can predict progression-free survival (PFS) and overall survival (OS) [12]. Moreover, Ben Bouallègue et al. demonstrated that heterogeneity parameters derived from the delineated MTV can serve as additional predictors of early metabolic response in adults with bulky Hodgkin and non-Hodgkin lymphomas [13].

Accordingly, the aim of this study was to identify metabolic or heterogeneity parameters from pretherapeutic FDG-PET/CT to predict inadequate response (IR) in pediatric patients with HL. PFS or OS were not selected as endpoints in this study because both are not only determined by initial characteristics/risk profile (which was the main focus of this study) but also by the extent of treatment (and namely the performance of RT).

## Methods

### Patients

This retrospective study included 50 consecutively examined children with classical HL (female, *n* = 18; male, *n* = 32; median age, 14.8 years; range, 4.0 to 18.0 years) treated according to EuroNet-PHL-C1 (*n* = 42) or -C2 treatment protocol (*n* = 8) between 2007 and 2017 [2, 3]. It included 31 patients with nodular sclerosing type and 17 patients with mixed cellularity type (not further specified, *n* = 2). These protocols are consecutive multinational standardized treatment protocols for pediatric patients with HL (lymphocyte-predominant subtype excluded). All patients undergo two cycles of induction chemotherapy (OEPA) before ERA with FDG-PET/CT is performed. This is followed by further chemotherapy (none in TG 1, one cycle in TL 1 with AR, two to four cycles in TG/TL 2 or 3) and, most importantly, determines the necessity of additional radiation therapy of the initially involved regions (all TG/TL in case of IR). In TG/TL 2 or 3, additional LRA is conducted after the second treatment segment in patients with IR at ERA to decide on further intensification of the subsequent radiation therapy [2, 3].

In patients diagnosed before November 2012, TG 1 included stage I and IIA without extranodal disease, TG 2 covered stage I or IIA with extranodal disease and any stage IIB or IIIA, and TG 3 included stage IIB or IIIA with extranodal disease and any stage IIIB or IV [2]. In patients diagnosed after November 2012, TL 1 covered patients with stage I and IIA without any risk factor while TL 2 included stage I or IIA with elevated ESR or bulk or extranodal disease and any stage IIB or IIIA without extranodal disease. TL 3 covered stage IIB or IIIA with extranodal disease and any stage IIIB or IV [2, 3].

### Positron emission tomography/computed tomography (PET/CT)

PET/CT imaging was performed using the tracer FDG and a dedicated PET/CT device (Gemini TF 16; Philips, Amsterdam, The Netherlands) with Philips Astonish TF technology. FDG was administered intravenously using a weight-adapted activity (median, 250 MBq; interquartile range [IQR], 170 to 275 MBq) based on recommendations provided by the European Association of Nuclear Medicine (EANM) [14]. A test of blood glucose level was mandatory to assure that blood glucose level was ≤8.3 mmol/l. The PET scan was performed after a median uptake time of 63 min (IQR, 57 to 76 min) in supine position from base of skull to the proximal femora with an axial field of view of 180 mm (3D mode; bed overlap, 53.3%). Attenuation correction was either based on contrast-enhanced CT (*n* = 33; automatic tube current modulation; weight-dependent maximum tube current, 100 to 200 mA; tube voltage, 120 kV; gantry rotation time, 0.5 s) or non-enhanced low-dose CT (*n* = 17; automatic tube current modulation; maximum tube current, 80 mA; tube voltage, 120 kV; gantry rotation time, 0.5 s). PET raw data was reconstructed iteratively with TOF analysis (BLOB-OS-TF; iterations, 3; subsets, 33; Philips Astonish TF technology). Projection data was reconstructed with 4 mm slice thickness (rows, 144; columns, 144; voxel size, 4x4x4 mm).

### Quantitative FDG-PET analysis

**Fig. 1:**
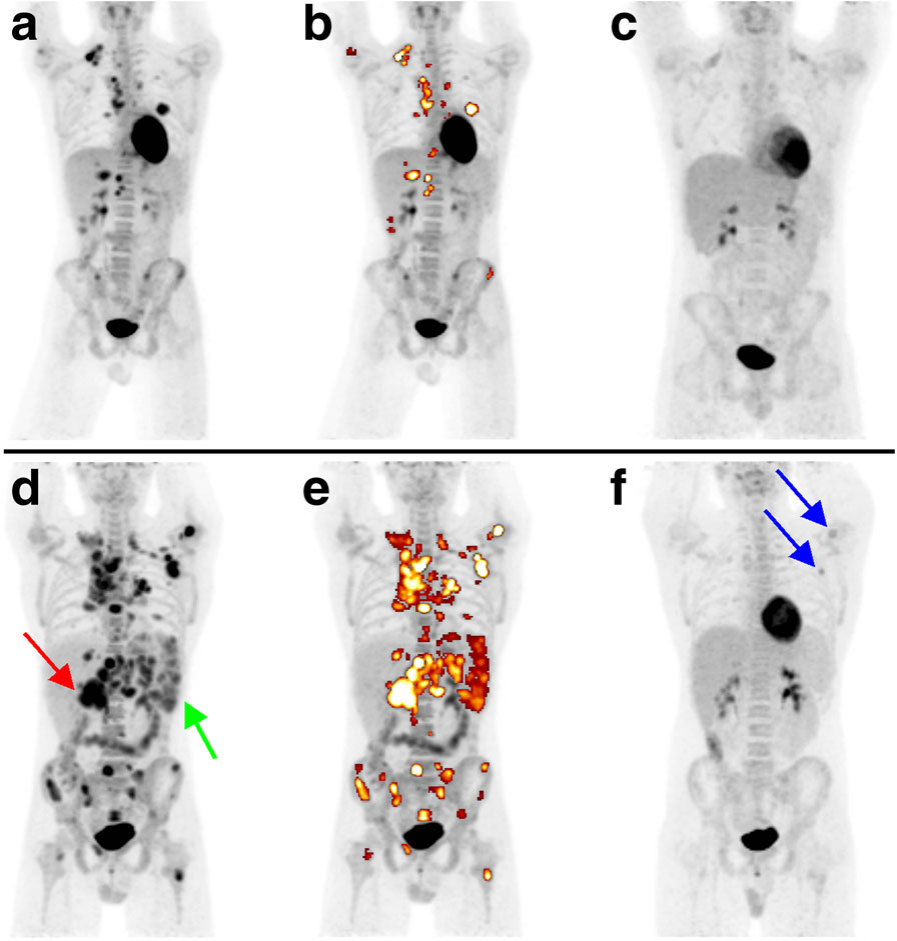
Patient examples of low and high MTV. Representative examples of FDG-PET maximum intensity projections (MIP) of two patients with stage IV disease before induction therapy (**a**, **b** + **d**, **e**) and at ERA (**c**, **f**). In the middle column (**b**, **e**), the delineated pretherapeutic MTV is colored (high activity: white, low activity: brownish). **a**-**c**: A 17-year-old male with stage IV disease (liver, lung) and AR who had a low MTV (51 ml). **d**-**f**: A 17-year-old male with stage IV disease (skeletal) and IR who showed a high MTV (792 ml); please also note the large lymph node mass at the liver hilus (*red arrow*) and extensive splenic involvement (*green arrow*). At ERA, considerable FDG uptake (Deauville score 4) can still be detected especially in a left axillary lymph node and the left humerus (*blue arrows*)

Quantification of FDG-PET data was performed with dedicated software (ROVER, version 3.0.34, ABX advanced biochemical compounds GmbH, Radeberg, Germany). All analysis was performed blinded to the results of ERA; however, the identification of HL lesions comprised all clinical and imaging data available in the pre-treatment setting including the final tumor stage as defined by interdisciplinary consensus (nuclear medicine physician, pediatric radiologist, pediatric oncologist, radiation oncologist, pediatric surgeon). The combined MTV of the entire FDG-avid HL lesion load of the patient (nodal and extranodal disease) was delineated using a semi-automatic, background-adapted algorithm [15, 16] (Fig. 1). The first step involved delineation on a per-lesion basis, visual inspection and manual correction if this deemed necessary. Manual correction (i.e. manually adjusted threshold or separate delineation of subvolumes) was necessary for 87 of 624 lesions (13.9%) affecting 26 of 50 patients. Manual correction was performed either for lesions with relatively low activity concentration compared to other lesions in the same body compartment that had to be delineated in a separate subvolume (83 of 624 lesions; 26 of 50 patients) or for lesions with highly heterogeneous intralesional activity concentration that required subdivision of the lesion (4 of 624 lesions; 3 of 50 patients). Bone marrow involvement was only diagnosed by PET if focal uptake could be clearly delineated.

After delineation of all individual lesions in one patient, the entirety of these lesional MTV was regarded as the patient’s total MTV which was exclusively used for final analysis and to derive all other parameters. As further metabolic parameters the SUVmax, SUVmean, SUVpeak (mean value of a spherical ROI with a diameter of approximately 1.2 cm centered at the ROI maximum) and total lesion glycolysis (TLG; MTV*SUVmean) were calculated for the whole MTV. Heterogeneity parameters were derived including the asphericity (ASP) [17, 18], entropy, energy, contrast, local homogeneity [19, 20], and cumulative SUV-volume histograms (CSH) [21, 22].

For comparative analysis (see Additional file 1), the MTV in all patients was also delineated with a fixed relative threshold of 41% of the maximum activity (MTV_t41_) and a fixed absolute threshold of SUV = 2.5 (MTV_2.5_).

### Early response assessment (ERA)

ERA was performed according to the respective treatment protocol after 2 cycles of induction chemotherapy.

In patients treated according to EuroNet-PHL-C1 protocol [2], IR was assigned if no overall complete response on morphological imaging was seen and any initially involved site was still PET-positive (based on International Harmonization Project criteria [23]); IR was also defined if no change was seen in morphological imaging (irrespective of PET) or if disease was still detectable on morphological imaging and PET was unclear. According to EuroNet-PHL-C2 protocol [3], IR in these patients was assigned if at least one site showed remaining FDG uptake higher than liver uptake on visual assessment (Deauville ≥ 4) or showed a qPET value ≥ 1.3 [6] or in case of poor bulk response (< 50% volume reduction) or if any nodal site with a diameter of ≥ 2 cm was nonassessable with qPET analysis. AR was assigned if IR criteria were not fulfilled and no disease progression was present. The assessment was verified by reference rating provided by the study group.

### Statistical analysis

Statistical analysis was performed using SPSS 22 (IBM Corporation, Armonk, NY, USA). Descriptive parameters were expressed as median, IQR and range or 95%-confidence interval (95%-CI), unless otherwise specified. Optimal cut-off values for quantitative FDG-PET parameters to distinguish IR from AR were defined by receiver operating characteristic (ROC) curves with respective areas under the curve (AUC). The optimal cut-off value was defined as the point on the ROC curve with the minimal distance *d* to the point (0,1) calculated as follows:

$$ d\kern0.5em =\kern0.5em \sqrt{{\left(1- Sensitivity\right)}^2\kern0.5em +\kern0.5em {\left(1- Specificity\right)}^2} $$

Patients were divided into groups of stages I/II versus III/IV and, alternatively, based on the assigned treatment group or treatment level (TG/TL) 1 versus 2 versus 3 according to the respective treatment protocol. Differences in MTV and ASP between these groups were investigated with Mann-Whitney *U* test. The relationship between a high MTV or high ASP, respectively, the patient’s tumor stage (I/II vs. III/IV) or TG/TL, and the result of ERA (IR vs. AR) was further assessed with log-linear analysis. Statistical significance was assumed at a *p* ≤ 0.05.

## Results

One of 50 patients had stage I, 26 patients had stage II, 7 patients had stage III, and 16 patients had stage IV disease (Table 1). Twelve patients were assigned TG/TL 1, 17 patients TG/TL 2, and 21 patients were assigned TG/TL 3. IR was observed in 28 of 50 patients, including 6 of 12 patients of TG/TL 1, 10 of 17 patients of TG/TL 2, and 12 of 21 patients of TG/TL 3.

**Table 1:** Patient characteristics

Parameter	All patients *(%)*	Patients with IR *(%)*	Patients with AR *(%)*
Total	50	28	22
Sex			
Female	18 (36)	12 (43)	6 (27)
Male	32 (64)	16 (57)	16 (73)
Stage			
I	1 (2)	0	1 (5)
II	26 (52)	16 (57)	10 (45)
III	7 (14)	2 (7)	5 (23)
IV	16 (32)	10 (36)	6 (27)
TG/TL			
1	12 (24)	6 (21)	6 (27)
2	17 (34)	10 (36)	7 (32)
3	21 (42)	12 (43)	9 (41)
ESR ≥ 30 mm/h			
Yes	33 (66)	19 (68)	14 (64)
No	17 (34)	9 (32)	8 (36)
Bulk ≥ 200 ml			
Yes	17 (34)	12 (43)	5 (23)
No	33 (66)	16 (57)	17 (77)
B-symptoms			
Yes	23 (46)	12 (43)	11 (50)
No	27 (54)	16 (57)	11 (50)
Extranodal			
Yes	8 (16)	4 (14)	4 (18)
No	42 (84)	24 (86)	18 (82)
Protocol			
PHL-C1	42 (84)	25 (89)	17 (77)
PHL-C2	8 (16)	3 (11)	5 (23)

*IR* inadequate response, *AR* adequate response, *TG/TL* treatment group/level, *ESR* erythrocyte sedimentation rate

### Metabolic and heterogeneity parameters in relation to stage and TG/TL

**Fig. 2:**
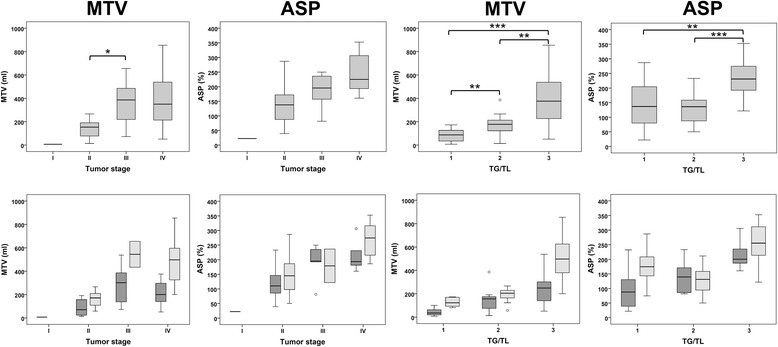
Box plots for MTV and ASP in different stages and TG/TL. In the upper row, box plots for MTV and ASP are separated only by different stages or TG/TL; significant differences between subgroups are highlighted (*p < 0.05; **p < 0.01; ***p < 0.001). Please note that only one patient had stage I disease which was therefore excluded from comparison. In the lower row, box plots are further separated by AR (*dark grey*) or IR (*light grey*); due to the smaller sample size, significance of the differences was not tested. ASP, asphericity; MTV, metabolic tumor volume, TG/TL, treatment group/level

Median MTV was 7.0 ml in stage I (one patient only), 154.0 ml (IQR, 73.9 to 194.2 ml) in stage II, 386.2 ml (137.9 to 537.8 ml) in stage III, and 350.6 ml (207.4 to 555.9 ml) in stage IV patients (Fig. 2) with significant differences only between stages II and III (*p* = 0.01). Comparison with stage I was not performed as it only included one patient. ASP in stage I was 22.2%, in stage II it was 137.9% (87.4 to 179.1%), in stage III 195.5% (121.7 to 236.4%), and in stage IV it was 224.9% (190.1 to 306.3%); no significant differences were detected. Among the remaining metabolic and heterogeneity parameters, only TLG was significantly different between stage II and III (*p* = 0.009).

The median MTV in TG/TL 1 was 87.4 ml (29.3 to 138.9 ml) compared to TG/TL 2 with 177.4 ml (103.4 to 221.0 ml) and TG/TL 3 with 375.8 ml (213.9 to 555.3 ml). MTV was significantly higher in TG/TL 3 vs. TG/TL 2 vs. TG/TL 1 patients (each *p* < 0.01). ASP in TG/TL 1 was 136.8% (77.4 to 206.3%) compared to 136.2% (91.2 to 165.3%) in TG/TL 2 (*p* = 0.95) and TG/TL 3 with 231.0% (189.7 to 290.3%). ASP in TG/TL 3 patients was significantly higher compared to TG/TL 1 or TG/TL 2 patients (each *p* < 0.01). Among the remaining parameters, a significant difference between TG/TL 1 vs. 2 was measured for SUVmax, SUVmean, SUVpeak, TLG, contrast, and local homogeneity (each *p* < 0.05). Significant differences between TG/TL 2 vs. 3 were observed for TLG, entropy, contrast, local homogeneity, energy, and CSH (each *p* < 0.05).

### Prediction of IR – different stages

MTV showed the highest AUC of all PET parameters; AUC for MTV in patients with stage I/II was 0.84 (95%-CI, 0.69 to 0.99), AUC in patients with stage III/IV was 0.86 (0.7 to 1.0). Using the respective optimal cut-off value (stage I/II, > 80 ml; stage III/IV, > 410 ml), patients with high vs. low MTV showed IR in 78.9 vs. 12.5% in stage I/II as well as 90.0 vs. 23.1% in stage III/IV. Sensitivity, specificity, negative predictive value (NPV), and positive predictive value (PPV) to predict IR were 94, 64, 88, and 79% in stage I/II compared to 75, 91, 77, and 90% in stage III/IV (Tables 2 and 3). Log-linear analysis showed a significant relationship between a high MTV and the response to induction therapy (IR vs. AR; z value, 3.9; *p* < 0.001) but not between tumor stage (I/II vs. III/IV) and MTV or response to therapy (both *p* > 0.05).

**Table 2:** Results of ROC analysis separated by stage

	Stage I/II		Stage III/IV	
	AUC (95%-CI)	Optimal cut-off value	AUC (95%-CI)	Optimal cut-off value
Metabolic parameters				
MTV	0.84 (0.69 to 0.99)	> 80 ml	0.86 (0.7 to 1.0)	> 410 ml
SUVmax	0.56 (0.33 to 0.8)	> 10.2	0.71 (0.5 to 0.93)	> 13.8
SUVmean	0.61 (0.39 to 0.83)	> 4.1	0.71 (0.5 to 0.93)	> 5.9
SUVpeak	0.58 (0.35 to 0.81)	> 9.1	0.74 (0.53 to 0.95)	> 12.6
TLG	0.77 (0.58 to 0.97)	> 450 ml	0.86 (0.7 to 1.0)	> 2800 ml
Heterogeneity parameters				
ASP	0.65 (0.43 to 0.88)	> 92%	0.74 (0.54 to 0.95)	> 210%
Entropy	0.58 (0.35 to 0.81)	< 5.82	0.58 (0.33 to 0.82)	< 5.73
Contrast	0.67 (0.45 to 0.89)	< 25	0.66 (0.43 to 0.89)	< 16
Local homogeneity	0.58 (0.33 to 0.83)	> 24	0.68 (0.45 to 0.91)	> 30.4
Energy	0.58 (0.34 to 0.82)	> 0.394	0.55 (0.31 to 0.8)	> 0.51
CSH	0.49 (0.25 to 0.74)	> 0.44	0.46 (0.21 to 0.7)	> 0.44

*MTV* metabolic tumor volume, *95%-CI* 95%-confidence interval, *TLG* total lesion glycolysis, *ASP* asphericity, *CSH* cumulative SUV-volume histograms

**Table 3:** Diagnostic accuracy of MTV and ASP towards IR (stages)

	Stage I/II		Stage III/IV	
Parameter	Sens. (95%-CI)	Spec. (95%-CI)	Sens. (95%-CI)	Spec. (95%-CI)
	NPV (95%-CI)	PPV (95%-CI)	NPV (95%-CI)	PPV (95%-CI)
MTV	94% (70 to 100%)	64% (31 to 89%)	75% (43 to 95%)	91% (59 to 100%)
	88% (47 to 100%)	79% (54 to 94%)	77% (46 to 95%)	90% (55 to 100%)
ASP	81% (54 to 96%)	55% (23 to 83%)	83% (52 to 98%)	64% (31 to 89%)
	67% (30 to 93%)	72% (47 to 90%)	78% (40 to 97%)	71% (42 to 92%)

*Sens.* sensitivity, *95%-CI* 95%-confidence interval, *spec.* Specificity, *NPV* negative predictive value, *PPV* positive predictive value, *MTV* metabolic tumor volume, *ASP* asphericity

Among heterogeneity parameters only, ASP provided the highest AUC in patients with stage I/II of 0.65 (0.43 to 0.88) and in stage III/IV of 0.74 (0.54 to 0.95; Tables 2 and 3). There was a significant relationship between high ASP and the response to induction therapy (z value, 2.8; *p* < 0.01) but not between tumor stage and ASP or response to therapy (both *p* > 0.05).

### Prediction of IR – different treatment groups/levels

The average AUC across all TG/TL was highest for MTV; AUC was 0.92 (0.74 to 1.0) for TG/TL 1, 0.71 (0.44 to 0.99) for TG/TL 2, and 0.85 (0.69 to 1.0) for TG/TL 3. Patients with high vs. low MTV had IR in 85.7 vs. 0% in TG/TL 1 (optimal cut-off, > 80 ml), 80.0 vs. 28.6% in TG/TL 2 (cut-off, > 160 ml), and 90.0 vs. 27.3% in TG/TL 3 (cut-off, > 410 ml). Sensitivity, specificity, NPV, and PPV of MTV to predict IR in TG/TL 1 were 100, 83, 100, and 86% compared to 80, 71, 71, and 80% in TG/TL 2 and 75, 89, 73, and 90% in TG/TL 3 (Tables 4 and 5). The relationship between high MTV and response to therapy was significant in log-linear analysis (z value, 3.7; *p* < 0.001) but not the relation between TG/TL and MTV or the response to therapy (both *p* > 0.05).

**Table 4:** Results of ROC analysis separated by TG/TL

	TG/TL 1		TG/TL 2		TG/TL 3	
	AUC (95%-CI)	Optimal cut-off value	AUC (95%-CI)	Optimal cut-off value	AUC (95%-CI)	Optimal cut-off value
Metabolic parameters						
MTV	0.92 (0.74 to 1.0)	> 80 ml	0.71 (0.44 to 0.99)	> 160 ml	0.85 (0.69 to 1.0)	> 410 ml
SUVmax	0.47 (0.1 to 0.86)	> 7.5	0.61 (0.29 to 0.94)	> 13.9	0.67 (0.43 to 0.91)	> 13.8
SUVmean	0.39 (0.0 to 0.76)	> 3.3	0.73 (0.49 to 0.97)	> 6.6	0.69 (0.46 to 0.93)	> 5.9
SUVpeak	0.42 (0.0 to 0.79)	> 5.3	0.6 (0.28 to 0.92)	> 12.3	0.71 (0.48 to 0.94)	> 12.6
TLG	0.89 (0.7 to 1.0)	> 320 ml	0.66 (0.38 to 0.93)	> 890 ml	0.85 (0.69 to 1.0)	> 2800 ml
Heterogeneity parameters						
ASP	0.78 (0.49 to 1.0)	> 137%	0.5 (0.2 to 0.8)	> 90%	0.7 (0.48 to 0.93)	> 235%
Entropy	0.61 (0.27 to 0.95)	< 5.9	0.53 (0.22 to 0.84)	< 5.82	0.51 (0.25 to 0.77)	< 5.73
Contrast	0.78 (0.51 to 1.0)	< 39	0.54 (0.24 to 0.85)	< 17.6	0.61 (0.35 to 0.87)	< 19
Local homogeneity	0.67 (0.34 to 1.0)	> 21.8	0.43 (0.11 to 0.75)	> 23.3	0.64 (0.38 to 0.9)	> 30.4
Energy	0.58 (0.23 to 0.93)	> 0.37	0.54 (0.23 to 0.85)	> 0.41	0.48 (0.22 to 0.75)	> 0.51
CSH	0.33 (0.0 to 0.67)	> 0.52	0.63 (0.32 to 0.94)	> 0.44	0.51 (0.25 to 0.77)	> 0.41

*TG/TL* treatment group / level, *95%-CI* 95%-confidence interval, *MTV* metabolic tumor volume, *TLG* total lesion glycolysis, *ASP* asphericity, *CSH* cumulative SUV-volume histograms

**Table 5:** Diagnostic accuracy of MTV and ASP towards IR (TG/TL)

	TG/TL 1		TG/TL 2		TG/TL 3	
Parameter	Sens. (95%-CI)	Spec. (95%-CI)	Sens. (95%-CI)	Spec. (95%-CI)	Sens. (95%-CI)	Spec. (95%-CI)
	NPV (95%-CI)	PPV (95%-CI)	NPV (95%-CI)	PPV (95%-CI)	NPV (95%-CI)	PPV (95%-CI)
MTV	100% (54 to 100%)	83% (36 to 100%)	80% (44 to 97%)	71% (29 to 96%)	75% (43 to 95%)	89% (52 to 100%)
	100% (48 to 100%)	86% (42 to 100%)	71% (29 to 96%)	80% (44 to 97%)	73% (39 to 94%)	90% (55 to 100%)
ASP	83% (36 to 100%)	67% (22 to 96%)	80% (44 to 97%)	43% (10 to 82%)	58% (28 to 85%)	78% (40 to 97%)
	80% (28 to 99%)	71% (29 to 96%)	60% (15 to 95%)	67% (35 to 90%)	58% (28 to 85%)	78% (40 to 97%)

*TG/TL* treatment group/level, *Sens.* sensitivity, *95%-CI* 95%-confidence interval, *spec.* Specificity, *NPV* negative predictive value, *PPV* positive predictive value, *MTV* metabolic tumor volume, *ASP* asphericity

Among the heterogeneity parameters, ASP provided the highest AUC in patients within TG/TL 1 of 0.78 (0.49 to 1.0) compared to 0.5 (0.2 to 0.8) in TG/TL 2 and to 0.7 (0.48 to 0.93) in TG/TL 3 (Tables 4 and 5). There was a significant relationship between high ASP and the response to therapy (z value, 2.6; *p* < 0.01) but not between TG/TL and ASP or response to therapy (both *p* > 0.05).

Patients with either high ASP or MTV vs. patients with both low MTV and ASP had IR in 85.7 vs. 0% (TG/TL 1), 71.2 vs. 0% (TG/TL 2), and 76.9 vs. 25% (TG/TL 3).

## Discussion

The aim of the present study was to investigate the predictive value of several quantitative parameters derived from pretherapeutic FDG-PET in pediatric HL regarding the occurrence of IR at ERA after induction therapy.

The total MTV of all nodal and extranodal HL lesions of the patients predicted IR with high but varying accuracy within all three TG/TL (AUC, 0.71 to 0.92). Furthermore, the optimal cut-off to distinguish patients with IR or AR was distinctly different between either stages I/II versus III/IV (cut-off, > 80 ml vs. > 410 ml) or between the three TG/TL (> 80 ml vs. > 160 ml vs. > 410 ml). This reflects an increasing average MTV especially between stage I/II and stage III/IV disease as one expects given the supposed increase in involved regions – while, interestingly, the average MTV in stage III and stage IV was similar (Fig. 2).

This study shows that quantitative parameters from pretherapeutic FDG-PET can predict IR to induction therapy in pediatric HL. This could help to further improve the established risk stratification. Patients with a high risk for IR could benefit from more intense induction therapy to increase their probability of achieving AR and avoid additional radiation therapy. Vice versa, patients with a priori especially low risk for IR might be the best candidates for the pursued treatment de-escalation. With regard to the patient’s outcome, Cottereau et al. demonstrated that the baseline MTV in adult patients with peripheral T-cell lymphoma (PTCL) helps to predict PFS and OS [24]. Both Cottereau et al. and Meignan et al. [12] delineated the MTV based on 41% of the maximum activity (i.e. t41 in the Supplementary material of the present study). However, Cottereau et al. further showed that a fixed threshold of 41% of maximum activity and different adaptive thresholds render highly correlative MTV and equally predict PFS and OS in patients with PTCL [25]. In the current study, the MTV was primarily defined using a background-adapted semi-automated algorithm (BG) but comparison with t41 confirmed this high inter-method agreement in measured MTV and ASP (see Additional file 1). In contrast, agreement with an absolute threshold of SUV = 2.5 was lower. However, larger patient samples would be required to evaluate if one of the methods is significantly more accurate to predict the patient’s outcome. In a study by Kanoun et al. with 59 mainly adult HL patients, MTV delineated with a 41% relative threshold or a fixed SUV of 2.5 equally allowed to identify patients with impaired PFS despite considerable inter-method MTV differences [26]. Nevertheless, advantageous practicality or robustness might favor one of the delineation approaches. Only BG and t41 allow for a differentiated delineation of lesions taking into account their intralesional and interlesional uptake heterogeneity – which is especially true for BG (see [16] for details). However, this might prolongate the delineation process if neighboring lesions within a subvolume (e.g. the mediastinum) are especially heterogeneous. An absolute SUV threshold disregards such heterogeneities which can facilitate lesion delineation despite the necessity to adjust the absolute SUV level in high background activity (e.g. spleen or bones) or to manually exclude background voxels from the MTV. For background-adapted MTV delineation, we used an algorithm developed at our site, but several other viable automated algorithms have been published [27–35]. It can be assumed that these algorithms perform similar to the algorithm used here.

Further metabolic parameters performed slightly (TLG) or considerably worse (different SUVs) compared to the MTV. This is in accordance with a study by Bouallègue et al. who did not find an association between SUVmax, SUVmean or SUVpeak with the early metabolic response in 57 patients (mostly adults) with HL or non-Hodgkin lymphoma [13]. The observation that the FDG uptake intensity in HL lesions is of less relevance than the anatomical distribution or volumetric extent of the lesions might be attributed to the multifocal/systemic nature of HL in contrast to other childhood malignancies such as Ewing’s sarcoma [36] or osteosarcoma [37] in which the pre-treatment SUVmax is a prognostic factor. Furthermore, the examined heterogeneity parameters – among which the ASP still performed best – showed lower predictive accuracy on average than the MTV. Except for the ASP, none of the heterogeneity parameters provided consistent predictive value sufficient for clinical application. Similarly, in the study by Ben Bouallègue et al., entropy, contrast and CSH were no significant predictors of early metabolic response (neither was the ASP) [13]. To estimate why certain heterogeneity parameters are especially relevant in different tumor entities requires thorough consideration of methodological (spatial resolution, voxel size), biological (lesion number and sizes), and metabolic features (intralesional heterogeneity of FDG uptake intensity). The examined heterogeneity parameters are differently susceptible to these factors. More specifically, the ASP is a priori independent not only from the size of the MTV itself but also from the heterogeneity of the intralesional activity distribution (it only depends on the MTV’s surface complexity). Thus, it could in principal be valuable as an additional quantitative parameter to the MTV to improve the predictive value of pretherapeutic PET in pediatric HL in all or only some of the different TG/TL. This combined risk assessment could especially help to increase the sensitivity to identify patients at risk for IR which is likely the primary clinical goal. Both parameters were significantly related to the response to induction therapy when evaluated in separate models (log-linear analysis); however, the evaluation of an independent predictive relevance of both parameters in a combined analysis would require a larger sample size. This is also true for the relationship between MTV and ASP with the tumor stage or TG/TL.

This retrospective explorative study is limited by the investigation of only 50 patients (although treated consecutively) which necessitates a larger study with an independent patient cohort to validate the presented results and to further elucidate the predictive value of single parameters within the relevant subgroups. Furthermore, as only one patient had stage I, no specific conclusions can be drawn for this stage in particular.

The response to induction therapy at ERA was used as endpoint in the current study but is only a surrogate for patient outcome – as opposed to the PFS or OS. Indeed, the predictive value of a high pretherapeutic MTV independent from the German Hodgkin Study Group (GHSG) risk group has been recently demonstrated retrospectively in 267 adult patients with early stage HL [38]. Nevertheless, one must be aware that a long-term endpoint as PFS or OS implies a certain bias of response-dependent treatment intensification (namely radiotherapy) that can only be avoided by a prospective study design or an early universal surrogate parameter such as the IR to a uniform induction treatment.

## Conclusions

In this explorative study, a high total MTV best predicted IR to induction therapy in pediatric HL of all pretherapeutic FDG-PET parameters. This was true in both low and high stages as well as the three different TG/TL. Among the investigated heterogeneity parameters, only ASP may be sufficiently predictive of IR to serve as a supplemental parameter to the MTV and further improve the predictive accuracy. The influence of inter-method MTV variability of different delineation approaches on their predictive accuracy requires further investigation.

## Additional file

Additional file 1: Supplemental materials. Results of additional analysis on delineation with a fixed relative threshold (MTV_t41_) and a fixed absolute threshold (MTV_2.5_) in comparison with the background-adapted semi-automated delineation presented in the main manuscript (MTV_BG_) [39]. (DOCX 3060 kb)

## Abbreviations

95%-CI: 95%-confidence interval
AR: Adequate response
ASP: Asphericity
AUC: Area under the curve
CSH: Cumulative SUV-volume histograms
EANM: European Association of Nuclear Medicine
ERA: Early response assessment
ESR: Erythrocyte sedimentation rate
f: Female
FDG-PET/CT: F18-fluorodesoxyglucose-positron emission tomography/computed tomography
GHSG: German Hodgkin Study Group
HL: Hodgkin’s lymphoma
IQR: Interquartile range
IR: Inadequate response
LRA: Late response assessment
m: Male
MIP: Maximum intensity projection
MTV: Metabolic tumor volume
NPV: Negative predictive value
OEPA: combined chemotherapy with vincristine, etoposide, prednisone, and doxorubicin
OS: Overall survival
PFS: Progression-free survival
PPV: Positive predictive value
PTCL: Peripheral T-cell lymphoma
ROC curve: Receiver operating characteristic curve
RT: Radiotherapy
Sens.: Sensitivity
Spec.: Specificity
SUV: Standardized uptake value
TG/TL: Treatment group/treatment level
TLG: Total lesion glycolysis

## References

[1] Ward E, DeSantis C, Robbins A, Kohler B, Jemal A (2014). Childhood and adolescent cancer statistics, 2014. CA Cancer J Clin.

[2] First International Inter-Group Study for Classical Hodgkin’s Lymphoma in Children and Adolescents. Final protocol version, 6^th^ amendment. ClinicalTrials.gov Identifier: NCT00433459. https://clinicaltrials.gov/ct2/show/study/NCT00433459. Accessed 03 Nov 2017.

[3] Second International Inter-Group Study for Classical Hodgkin Lymphoma in Children and Adolescents. Final protocol version, 1^st^ amendment. NCT02684708. https://clinicaltrials.gov/ct2/show/NCT02684708. Accessed 03 Nov 2017.

[4] Barrington SF, Qian W, Somer EJ, Franceschetto A, Bagni B, Brun E (2010). Concordance between four European centres of PET reporting criteria designed for use in multicentre trials in Hodgkin lymphoma. Eur J Nucl Med Mol Imaging.

[5] Furth C, Steffen IG, Amthauer H, Ruf J, Misch D, Schönberger S (2009). Early and late therapy response assessment with [18F]fluorodeoxyglucose positron emission tomography in pediatric Hodgkin’s lymphoma: analysis of a prospective multicenter trial. J Clin Oncol.

[6] Hasenclever D, Kurch L, Mauz-Körholz C, Elsner A, Georgi T, Wallace H (2014). qPET - a quantitative extension of the Deauville scale to assess response in interim FDG-PET scans in lymphoma. Eur J Nucl Med Mol Imaging.

[7] Furth C, Amthauer H, Hautzel H, Steffen IG, Ruf J, Schiefer J (2011). Evaluation of interim PET response criteria in paediatric Hodgkin's lymphoma--results for dedicated assessment criteria in a blinded dual-Centre read. Ann Oncol.

[8] Bhatia S, Yasui Y, Robinson LL, Birch JM, Bogue MK, Diller L (2003). High risk of subsequent neoplasms continues with extended follow-up of childhood Hodgkin’s disease: report from the late effects study group. J Clin Oncol.

[9] Morton LM, Onel K, Curtis RE, Hungate EA, Armstrong GT. The rising incidence of second cancers: patterns of occurrence and identification of risk factors for children and adults. Am Soc Clin Oncol Educ Book. 2014:e57-e67; doi:10.14694/EdBook_AM.2014.34.e57. 10.14694/EdBook_AM.2014.34.e57 24857148

[10] Bakhshi S, Bhethanabhotla S, Kumar R, Agarwal K, Sharma P, Thulkar S (2017). Posttreatment PET/CT rather than interim PET/CT using deauville criteria predicts outcome in pediatric hodgkin lymphoma: a prospective study comparing PET/CT with conventional imaging. J Nucl Med.

[11] Punwani S, Taylor SA, Saad ZZ, Bainbridge A, Groves A, Daw S (2013). Diffusion-weighted MRI of lymphoma: prognostic utility and implications for PET/MRI?. Eur J Nucl Med Mol Imaging.

[12] Meignan M, Cottereau AS, Versari A, Chartier L, Dupuis J, Boussetta S (2016). Baseline metabolic tumor volume predicts outcome in high-tumor-burden follicular lymphoma: a pooled analysis of three multicenter studies. J Clin Oncol.

[13] Ben Bouallègue F, Tabaa YA, Kafrouni M, Cartron G, Vauchot F, Mariano-Goulart D (2017). Association between textural and morphological tumor indices on baseline PET-CT and early metabolic response on interim PET-CT in bulky malignant lymphomas. Med Phys.

[14] Dosage card Version 5.7.2016. European Association of Nuclear Medicine. http://www.eanm.org/content-eanm/uploads/2017/01/EANM_Dosage_Card_040214.pdf. Accessed 27 Oct 2017.

[15] Hofheinz F, Poetzsch C, Oehme L, Beuthien-Baumann B, Steinbach J, Kotzerke J (2012). Automatic volume delineation in oncological PET. Evaluation of a dedicated software tool and comparison with manual delineation in clinical data sets. Nuklearmedizin.

[16] Hofheinz F, Langner J, Petr J, Beuthien-Baumann B, Steinbach J, Kotzerke J (2013). An automatic method for accurate volume delineation of heterogeneous tumors in PET. Med Phys.

[17] Apostolova I, Steffen IG, Wedel F, Lougovski A, Marnitz S, Derlin T (2014). Asphericity of pretherapeutic tumor FDG uptake provides independent prognostic value in head-and-neck cancer. Eur Radiol.

[18] Hofheinz F, Lougovski A, Zöphel K, Hentschel M, Steffen IG, Apostolova I (2015). Increased evidence for the prognostic value of primary tumor asphericity in pretherapeutic FDG PET for risk stratification in patients with head and neck cancer. Eur J Nucl Med Mol Imaging.

[19] Hatt M, Majdoub M, Vallières M, Tixier F, Le Rest CC, Groheux D (2015). 18F-FDG PET uptake characterization through texture analysis: investigating the complementary nature of heterogeneity and functional tumor volume in a multi-cancer site patient cohort. J Nucl Med.

[20] Vallières M, Freeman CR, Skamene SR, El Naqa I (2015). A radiomics model from joint FDG-PET and MRI texture features for the prediction of lung metastases in soft-tissue sarcomas of the extremities. Phys Med Biol.

[21] El Naqa I, Grigsby P, Apte A, Kidd E, Donnelly E, Khullar D (2009). Exploring feature-based approaches in PET images for predicting cancer treatment outcomes. Pattern Recogn.

[22] van Velden FH, Cheebsumon P, Yaqub M, Smit EF, Hoekstra OS, Lammertsma AA (2011). Evaluation of a cumulative SUV-volume histogram method for parameterizing heterogeneous intratumoural FDG uptake in non-small cell lung cancer PET studies. Eur J Nucl Med Mol Imaging.

[23] Cheson BD (2007). The international harmonization project for response criteria in lymphoma clinical trials. Hematol Oncol Clin North Am.

[24] Cottereau AS, El-Galaly TC, Becker S, Broussais F, Peterson LJ, Bonnet C, et al. Predictive value of PET response combined with baseline metabolic tumor volume in peripheral T-cell lymphoma patients. J Nucl Med. 2017. 10.2967/jnumed.117.193946. [Epub ahead of print]. 10.2967/jnumed.117.193946 28864629

[25] Cottereau AS, Hapdey S, Chartier L, Modzelewski R, Casasnovas O, Itti E (2017). Baseline total metabolic tumor volume measured with fixed or different adaptive thresholding methods equally predicts outcome in peripheral T cell lymphoma. J Nucl Med.

[26] Kanoun S, Tal I, Berriolo-Riedinger A, Rossi C, Riedinger JM, Vrigneaud JM (2015). Influence of software tool and methodological aspects of total metabolic tumor volume calculation on baseline [18F] FDG PET to predict survival in hodgkin lymphoma. PLoS One.

[27] Black QC, Grills IS, Kestin LL, Wong CY, Wong JW, Martinez AA (2004). Defining a radiotherapy target with positron emission tomography. Int J Radiat Oncol Biol Phys.

[28] Boellaard R, Krak NC, Hoekstra OS, Lammertsma AA (2004). Effects of noise, image resolution, and ROI definition on the accuracy of standard uptake values: a simulation study. J Nucl Med.

[29] Daisne JF, Sibomana M, Bol A, Doumont T, Lonneux M, Gregoire V (2003). Tri-dimensional automatic segmentation of PET volumes based on measured source-to-background ratios: influence of reconstruction algorithms. Radiother Oncol.

[30] Drever L, Robinson DM, McEwan A, Roa W (2006). A local contrast based approach to threshold segmentation for PET target volume delineation. Med Phys.

[31] Erdi YE, Mawlawi O, Larson SM, Imbriaco M, Yeung H, Finn R (1997). Segmentation of lung lesion volume by adaptive positron emission tomography image thresholding. Cancer.

[32] Frings V, de Langen AJ, Smit EF, van Velden FH, Hoekstra OS, van Tinteren H (2010). Repeatability of metabolically active volume measurements with 18F-FDG and 18F-FLT PET in non-small cell lung cancer. J Nucl Med.

[33] Jentzen W, Freudenberg L, Eising EG, Heinze M, Brandau W, Bockisch A (2007). Segmentation of PET volumes by iterative image thresholding. J Nucl Med.

[34] van Dalen JA, Hoffmann AL, Dicken V, Vogel WW, Wiering B, Ruers TJ (2007). A novel iterative method for lesion delineation and volumetric quantification with FDG PET. Nucl Med Commun.

[35] Vauclin S, Doyeux K, Hapdey S, Edet-Sanson A, Vera P, Gardin I (2009). Development of a generic thresholding algorithm for the delineation of 18FDG-PET-positive tissue: application to the comparison of three thresholding models. Phys Med Biol.

[36] Hwang JP, Lim I, Kong CB, Jeon DG, Byun BH (2016). Prognostic value of SUVmax measured by pretreatment Fluorine-18 Fluorodeoxyglucose positron emission tomography/computed tomography in patients with Ewing sarcoma. PLoS One.

[37] Costelloe CM, Macapinlac HA, Madewell JE, Fitzgerald NE, Mawlawi OR, Rohren EM (2009). 18F-FDG PET/CT as an indicator of progression-free and overall survival in osteosarcoma. J Nucl Med.

[38] Akhtari M, Milgrom SA, Pinnix CC, Reddy JP, Dong W, Smith GL (2018). Reclassifying patients with early-stage Hodgkin lymphoma based on functional radiographic markers at presentation. Blood.

[39] Shrout PE, Fleiss JL (1979). Intraclass correlations: uses in assessing rater reliability. Psychol Bull.

