# Supplementary material for: Pretherapeutic FDG-PET total metabolic tumor volume predicts response to induction therapy in pediatric Hodgkin’s lymphoma
Source: BMC Cancer. 2018 May 3;18:521. doi: 10.1186/s12885-018-4432-4 (PMC5934894; doi:10.1186/s12885-018-4432-4)
Supplement: Supplementary file 1 — Supplemental materials. Results of additional analysis on delineation with a fixed relative threshold (MTVt41) and a fixed absolute threshold (MTV2.5) in comparison with the background-adapted semi-automated delineation presented in the main manuscript (MTVBG) [39]. (DOCX 3060 kb) [file 12885_2018_4432_MOESM1_ESM.docx]

**Additional file 1**

**Supplemental Methods**

**Quantitative FDG-PET analysis**

In addition to the semi-automatic, background-adapted algorithm (MTV_BG_; see Methods section of the main manuscript), all lesions were also delineated with a fixed relative threshold of 41% of the maximum activity (non-background-adapted; MTV_t41_) and using a fixed absolute threshold of SUV = 2.5 (MTV_2.5_; Supplemental Figure 1). Again, manual correction was performed either for lesions with relatively low activity concentration compared to other lesions in the same body compartment that had to be delineated in a separate subvolume (MTV_t41_: 125 of 624 lesions; 32 of 50 patients; MTV_2.5_: 4 of 624 lesions; 3 of 50 patients) or for lesions with highly heterogeneous intralesional activity concentration that required subdivision of the lesion (MTV_t41_: 37 of 624 lesions; 26 of 50 patients). For MTV_2.5_ delineation, the absolute SUV threshold had to be increased (up to SUV = 5.5) in 17 patients to enable lesion delineation, and manual exclusion of physiological background activity was necessary in 88 of 624 lesions (34 of 50 patients).

**Statistical analysis**

Correlation between MTV_BG_, MTV_t41_, and MTV_2.5_ as well as between ASP_BG_, ASP_t41_, and ASP_2.5_ was determined by intraclass correlation ICC(2,k) according to Shrout and Fleiss [39] (absolute agreement; ICC for single measures). Agreement of these parameters was displayed using Bland-Altman plots and the 95%-limits of agreement (95%-LoA). Statistical significance was again assumed at a *p* ≤ 0.05.

**Supplemental Results**

**Correlation and agreement between different delineation approaches**

ICC of MTV_BG_ vs. MTV_t41_ was 0.95 (95%-CI, 0.91 to 0.98), ICC between MTV_BG_ and MTV_2.5_ was 0.79 (0.36 to 0.91). The mean absolute difference between MTV_t41_ minus MTV_BG_ was -15.6 ml (95%-LoA, -131.7 to +100.4 ml; Supplemental Figure 2) while the difference of MTV_2.5_ minus MTV_BG_ measured +119.8 ml (-161.9 to +401.4 ml).

ICC of ASP_BG_ and ASP_t41_ was 0.97 (95%-CI, 0.94 to 0.98) while ICC of ASP_BG_ and ASP_2.5_ was 0.83 (0.15 to 0.94). The mean absolute difference between ASP_t41_ minus ASP_BG_ was -6.8% (95%-LoA, -41.5 to +27.9%; Supplemental Figure 3) compared to a difference of ASP_2.5_ minus ASP_BG_ of -36.2% (-96.0 to +23.6%).

**Comparative predictive accuracy of the different MTV**

Optimal cut-offs to predict IR in TG/TL 1, 2, and 3 with MTV_BG_ were >80, >160, and >410 ml (see Results section in the main manuscript), for MTV_t41_, >75, >160, and >350 ml were optimal, and for MTV_2.5_, >125, >278, and >630 ml were optimal cut-off values.

MTV_BG_ and MTV_t41_ were concordantly correct to predict IR in 39 of 50 patients while both were incorrect in 9 patients, and only MTV_BG_ was correct in the remaining 2 patients (4%; Supplemental Table 1). Concordant correct or incorrect predictions by MTV_BG_ and MTV_2.5_ were observed in 37 and 7 patients, respectively, while only MTV_BG_ was correct in 4 patients (8%) and only MTV_2.5_ in 2 of 50 patients (4%).

**Comparative predictive accuracy of the different ASP**

Optimal cut-offs for prediction of IR in TG/TL 1, 2, and 3 with ASP_BG_ were >137, >90, and >235% (see Table 4 in the main manuscript), for ASP_t41_, >115, >95, and >196% were optimal, and for ASP_2.5_, >130, >88, and >187% were optimal cut-off values.

ASP_BG_, ASP_t41_ and ASP_2.5_ correctly predicted IR in 30 of 50 patients while all were incorrect in 11 patients, and only ASP_BG_ was correct in 5 patients (10%) compared to 4 patients (8%) correctly predicted only by ASP_t41_ and ASP_2.5_ (Supplemental Table 1).

**Supplemental Tables**

**TABLE 1.** Contingency tables to predict IR by MTV and ASP of the different delineation approaches

|  |  | **MTV_BG_** | |  |  |  | |  | **MTV_BG_** | |  |
| --- | --- | --- | --- | --- | --- | --- | --- | --- | --- | --- | --- |
|  |  | **Incorrect** | **Correct** | **Total** |  |  |  | | **Incorrect** | **Correct** | **Total** |
| **MTV_t41_** | **Incorrect** | 9 | 2 | *11* |  | **MTV_2.5_** | **Incorrect** | | 7 | 4 | *11* |
|  | **Correct** | 0 | 39 | *39* |  |  | **Correct** | | 2 | 37 | *39* |
|  | **Total** | *9* | *41* | *50* |  |  | **Total** | | *9* | *41* | *50* |
|  |  |  |  |  |  |  |  | |  |  |  |
|  |  | **ASP_BG_** | |  |  |  | |  | **ASP_BG_** | |  |
|  |  | **Incorrect** | **Correct** | **Total** |  |  |  | | **Incorrect** | **Correct** | **Total** |
| **ASP_t41_** | **Incorrect** | 11 | 5 | *16* |  | **ASP_2.5_** | **Incorrect** | | 11 | 5 | *16* |
|  | **Correct** | 4 | 30 | *34* |  |  | **Correct** | | 4 | 30 | *34* |
|  | **Total** | *15* | *35* | *50* |  |  | **Total** | | *15* | *35* | *50* |

MTV, metabolic tumor volume; ASP; asphericity.

**Supplemental Figures**

**FIGURE 1.** Patient example of the different MTV


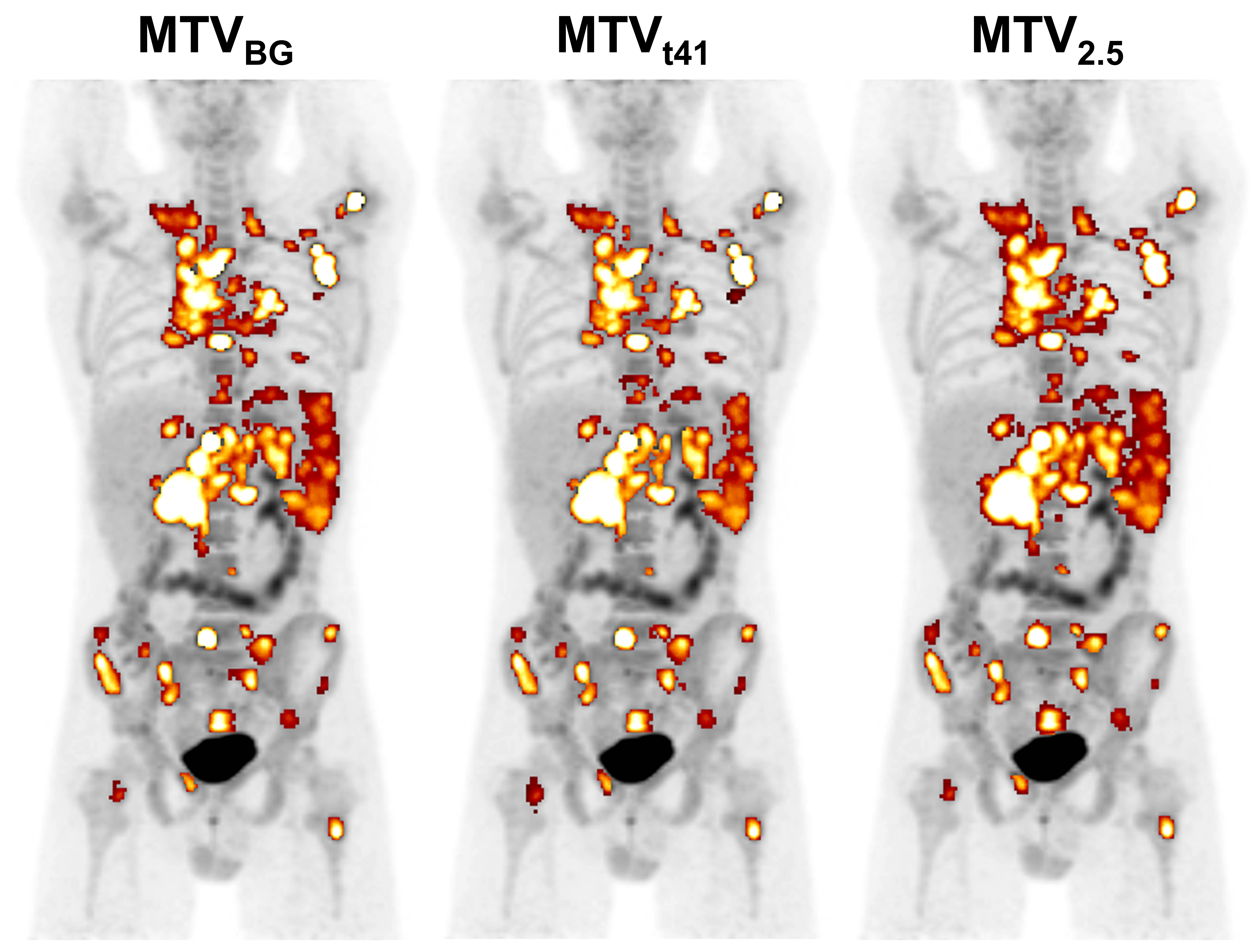


The same 17-year-old male patient as in Figure 1 in the main manuscript with stage IV disease (skeletal) who presented with IR and showed a high MTV_BG_ (792 ml). MTV_t41_ was 623 ml, and MTV_2.5_ was 1152 ml.

**FIGURE 2.** Bland-Altman plots for MTV of the different delineation approaches


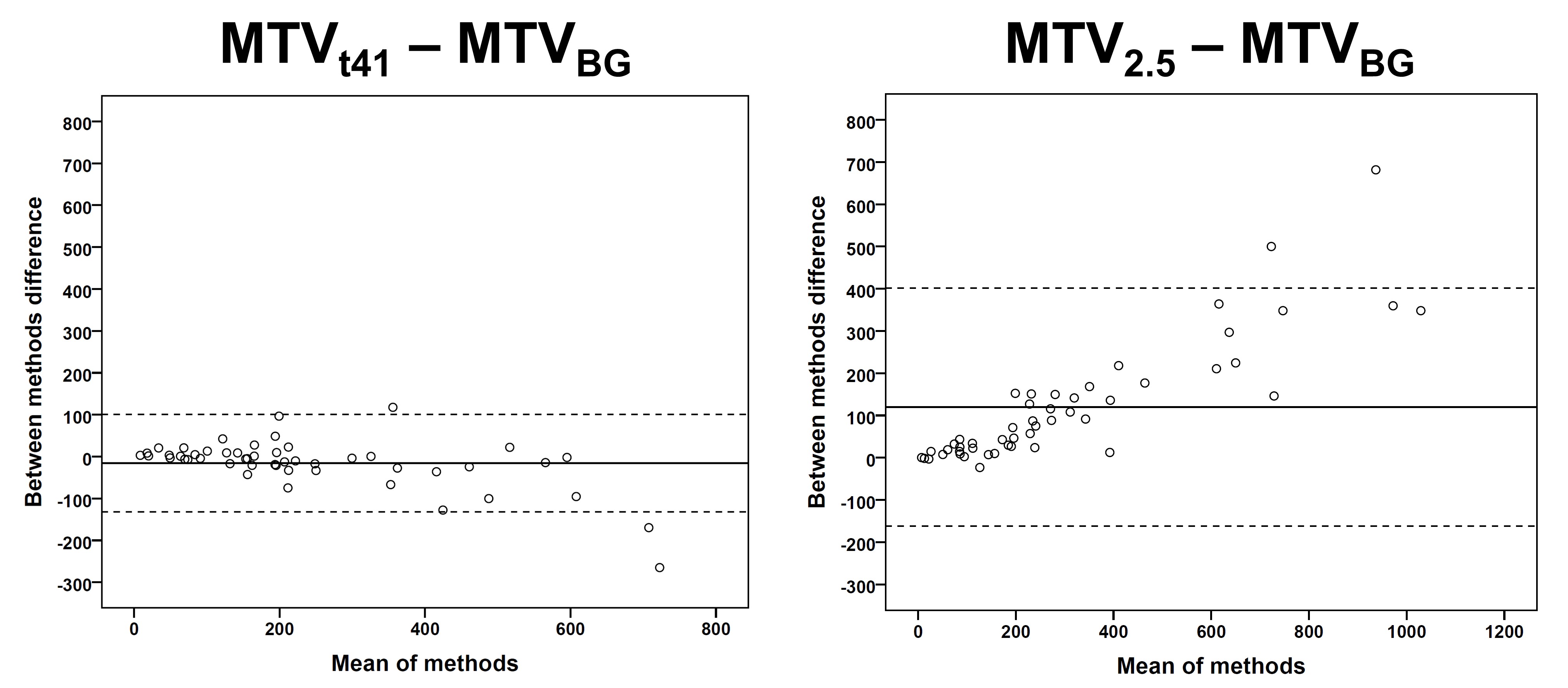


Agreement between MTV_t41_ and MTV_BG_ as well as MTV_2.5_ and MTV_BG_ is visualized with Bland-Altman plots including the mean absolute difference (solid line) and respective 95%-LoA (dashed lines).

**FIGURE 3.** Bland-Altman plots for ASP of the different delineation approaches


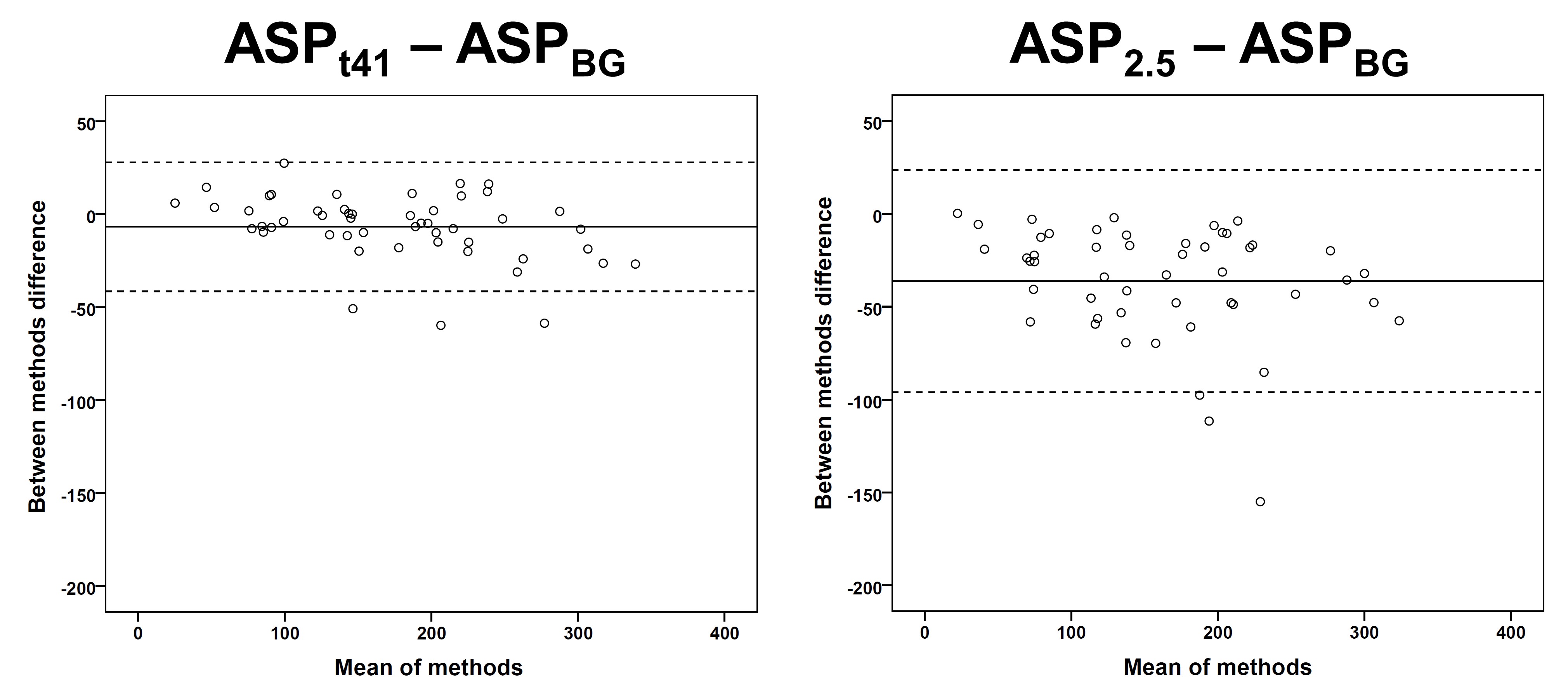


Agreement between ASP_t41_ and ASP_BG_ as well as ASP_2.5_ and ASP_BG_ is visualized with Bland-Altman plots including the mean absolute difference (solid line) and respective 95%-LoA (dashed lines).
